# Protective Effect of Natural Medicinal Plants on Cardiomyocyte Injury in Heart Failure: Targeting the Dysregulation of Mitochondrial Homeostasis and Mitophagy

**DOI:** 10.1155/2022/3617086

**Published:** 2022-09-12

**Authors:** Qi Wang, Hao Su, Jinfeng Liu

**Affiliations:** ^1^First Affiliated Hospital, Heilongjiang University of Chinese Medicine, Harbin 150040, China; ^2^Guang'anmen Hospital, Chinese Academy of Traditional Chinese Medicine, Beijing 100053, China

## Abstract

Heart failure occurs because of various cardiovascular pathologies, such as coronary artery disease or cardiorenal syndrome, eventually reaching end-stage disease. Various factors contribute to cardiac structural or functional changes that result in systolic or diastolic dysfunction. Several studies have confirmed that the key factor in heart failure progression is myocardial cell death, and mitophagy is the major mechanism regulating myocardial cell death in heart failure. The clinical mechanisms of heart failure are well understood in practice. However, the essential role of mitophagic regulation in heart failure has only recently received widespread attention. Receptor-mediated mitophagy is involved in various mitochondrial processes like oxidative stress injury, energy metabolism disorders, and calcium homeostasis, which are also the main causes of heart failure. Understanding of the diverse regulatory mechanisms in mitophagy and the complexity of its pathophysiology in heart failure remains incomplete. Related studies have found that various natural medicinal plants and active ingredients, such as flavonoids and saponins, can regulate mitophagy to a certain extent, improve myocardial function, and protect myocardial cells. This review comprehensively covers the relevant mechanisms of different types of mitophagy in regulating heart failure pathology and controlling mitochondrial adaptability to stress injury. Further, it explores the relationship between mitophagy and cardiac ejection dysfunction. Natural medicinal plant-targeted regulation strategies and scientific evidence on mitophagy were provided to elucidate current and potential strategies to apply mitophagy-targeted therapy for heart failure.

## 1. Introduction

Heart failure refers to a syndrome in which cardiac output is reduced because of primary heart damage and cannot meet the needs of tissue metabolism under normal venous return [1]. Clinically, it is typically characterized by systemic circulatory congestion of various etiologies representing the end-stage cardiac pathology [2]. The concepts of congestive heart failure and cardiac insufficiency (cardiac dysfunction) are the same; however, the latter has a broader meaning, including a stage of reduced cardiac output without clinical symptoms [3].

The key processes resulting in heart failure progression are myocardial cell death or severe ischemia and hypoxic injury, and autophagy is the main mechanism of cardiomyocyte death. Heart failure symptoms may be caused or aggravated by certain factors [4]: carditis or systemic infection; atrial fibrillation or various arrhythmias; water and electrolyte imbalance, including excessive sodium and abnormal fluid metabolism; physical exertion, mental stress, and emotional agitation; rapid changes in the environment and climate; increased cardiac load, such as that observed during pregnancy and childbirth; improper treatment, etc. [5]. Dysfunction of myocardial cell energy metabolism and insufficient energy supply are among the main mechanisms of heart failure and functional cardiac damage. Mitochondria are the primary sites of energy production in eukaryotic cells and provide adenosine triphosphate (ATP) to the body through oxidative phosphorylation. This maintains the basal metabolism of cardiomyocytes [6]. External stimuli or pathological conditions can impair mitochondrial structure and function, ultimately resulting in restricted ATP synthesis. Mitochondrial autophagy can selectively remove dysfunctional mitochondria, maintain the mitochondrial balance in cells, preserve the integrity of structure and function, and promote cell survival [7].

Abnormal mitophagy can lead to energy metabolism disorders that are closely associated with several symptoms accompanying heart failure [8]. Research related to mitophagy and energy metabolism has become the main investigative focus of heart failure's pathological and treatment mechanisms. It is important to explore the mechanisms of mitophagy and their relationship with heart failure caused by myocardial injury [9]. Moreover, mitophagy has a bidirectional regulatory effect on the physiological and pathological regulation of cardiomyocytes as well as can accurately and effectively intervene in the survival process of cardiomyocytes [10].

Research on mitochondrial thermoregulation by active ingredients in natural medicinal plants has developed rapidly [11]. In vivo and in vitro experimental studies have shown that the active ingredients in natural medicinal plants can affect mitophagy through receptor and nonreceptor regulation [9, 11, 12]. Thus, mitophagy and its signaling pathways may be important as preventive or therapeutic targets, for which the active components in natural medicinal plants appear to play an effective role at the molecular level [11, 13]. This review begins with the pathophysiology of heart failure and the regulatory processes of mitophagy. It expounds on the main regulatory receptor pathways of mitophagy and the role of natural medicinal plants in mitophagy within the pathological mechanism of heart failure. This review provides references and ideas for future related research.

## 2. Physiological and Pathological Mechanisms of Heart Failure

### 2.1. Structural Myocardial Damage or Ventricular Remodeling

Ventricular remodeling, caused by severe underlying diseases, myocardial infarction, or myocardial ischemia/reperfusion injury, is the most important pathological cause of heart failure [14]. The initial myocardial injury subsequently develops to myocardial fibrosis and cardiomyocyte hypertrophy, leading to ventricular remodeling and muscle hypertrophy, followed by cardiac chamber enlargement, and ultimately resulting in cardiac hemodynamic disturbances and heart failure [15, 16]. The mechanisms of pathological ventricular remodeling include cardiomyocyte hypertrophy, changes in the myocardial extracellular matrix, and profibrotic factors induced by various stress stimuli. Primary myocardial structural damage and cardiac overload can increase ventricular wall stress [17]. When myocardial hypertrophy can overcome the increased ventricular wall stress caused by the hypertrophy and fibrosis, ventricular function is maintained. This is the adaptive cardiac stage, when clinical symptoms of severe congestive heart failure do not occur [18]. When the myocardial hypertrophy is insufficient to overcome the increased ventricular wall stress, the heart will enter a period of maladaptation. At that time, the left ventricle will enlarge progressively with cardiac ejection dysfunction, which may eventually progress to irreversible myocardial damage. In the final stage, the hypertrophic myocardium has decreased systolic velocity and prolonged myocardial contraction time, but myocardial fiber shortening capacity and ventricular emptying capacity are not impaired [19]. Therefore, cardiac hypertrophy appears to play a beneficial compensatory role during the initial stages.

The mechanism by which a hypertrophic heart develops into progressive ventricular enlargement and heart failure is not well understood, and myocardial energy depletion may be a main causative factor. Because the hypertrophic myocardium is in a state of energy starvation, myocardial ischemia and myocardial cell death, followed by fibrosis further increase the load on the remaining viable myocardium. This, in turn, yields further myocardial hypertrophy with progressive fibrosis [20]. It all creates a vicious circle of energy metabolism disorders that may represent the mechanism that initiates ventricular remodeling.

### 2.2. Insufficient Myocardial Energy Supply and Abnormal Gene Expression

Mitochondria, the energy metabolism center of cardiomyocytes, may impact the regulatory mechanism of ventricular remodeling in heart failure. The heart has the greatest energy demand among various organs in the human body. When metabolic diseases, underlying diseases, and various myocardial ischemia/hypoxia-induced cardiac energy metabolic disorders occur, serious disturbances in the heart's biochemical regulatory mechanisms also occur [21]. These mainly involve excessive activation of the cardiac neuroendocrine hormones, changes in the adrenergic receptor system, and decreased synthesis of cyclic adenosine monophosphate. They lead to abnormalities in the calcium transport system, a disturbance of troponin's ability to bind calcium, and direct effects of endothelin and oxygen free radicals on the myocardium [22]. Oxidative stress, calcium overload, and mitochondrial injury jointly cause deficiencies in the myocardial energy supply chain and decrease myocardial energy utilization [23]. Myocardial hypertrophy in heart failure, a decrease in capillary density and mitochondrial number per unit area, and an increase in connective tissue in the myocardium and its periphery and the sinoatrial node can also cause myocardial cells to remain in a state of insufficient energy supply [24]. This study suggests that insufficient myocardial energy metabolism results in abnormal growth of the overloaded myocardium, thereby changing the normal expression of genes related to myocardial protein synthesis. Thus, the overloaded myocardium will accelerate myocardial failure under regulation [25].

### 2.3. Cardiac Hemodynamic Abnormalities

Hemodynamic abnormalities, caused by ischemia/reperfusion, myocardial infarction, cardiac fibrosis, myocardial hypertrophy, hypertension, pulmonary hypertension, and other causes, are among the predominant causes of heart failure [26]. To a certain extent, the ventricular function curve reflects the relationship between cardiac output and ventricular filling pressure [27]. On the aggravation of myocardial hypertrophy and myocardial fibrosis as well as the increase in ventricular filling pressure and end-diastolic length, stroke volume increases accordingly. When the stroke volume increases to a certain limit and the cardiac output reaches the maximum effect, the stroke volume does not increase further, but decreases [28]. Therefore, a decline phase occurs after the ventricular function curve has reached the plateau phase. Signs and symptoms of severely reduced cardiac output are present at this stage, leading to increased ventricular end-diastolic blood pressure [29]. The excessively elevated pulmonary venous pressure and capillary blood pressure can indirectly cause the symptoms and signs of pulmonary circulation congestion.

### 2.4. Activation of the Neuroendocrine System

Heart failure can lead to sympathetic dysfunction, and low cardiac output excites baroreceptors, resulting in sympathetic reflex activation. Early nervous system activation also increases myocardial contractility and cardiac output [30]. The regulation of peripheral vascular resistance to meet the needs of metabolism and vital organs is a means of hemodynamic compensation for the failing myocardium. However, excessive activation of the sympathetic nervous system can also result in myocardial hypertrophy, apoptosis, and interstitial fibrosis, thereby accelerating the deterioration of heart failure and inducing sudden death [31].

When heart failure occurs, the renin-angiotensin-aldosterone system is activated more slowly than the sympathetic nervous system [32]. Angiotensin receptor-II is the main active substance that is stimulated. It has a strong vasoconstrictive effect and creates increased ventricular afterload, resulting in myocardial cell hypertrophy, apoptosis, interstitial fibrosis, and ventricular and vascular remodeling [33].

Moreover, the renin-angiotensin-aldosterone system is closely related to the sympathetic nervous system, and sympathetic activation can increase renin release. Angiotensin receptor-II acts on the sympathetic nervous system, and positive feedback promotes the release of epinephrine, vasopressin, and aldosterone. Hyperaldosteronemia can in turn cause autonomic dysfunction, sympathetic activation, and decreased parasympathetic activity, particularly when there is myocardial extracellular matrix remodeling.

### 2.5. Changes in Myocardial Receptors and Oxidative Stress Injury

Abnormal regulation of myocardial *β*-receptors is one of the important factors leading to heart failure or disease progression. Long-term sympathetic excitation due to modulation of the neuroendocrine system as well as cardiac enlargement and hypertrophy, can decrease the density of *β*-receptors on the membrane of ventricular myocytes [34]. The cAMP content in myocardial cells decreases, and the contractile response to *β*-adrenergic stimulation is weakened; therefore, recovering the myocardial contractile function is difficult [35]. In addition, oxygen free radicals and ischemia accompanying heart failure can cause cardiac ejection dysfunction, while superoxide dismutase (SOD) and cholesterol acyltransferase (CAT) can promote cardiac function recovery [36]. The mechanisms of oxidative stress and antioxidant enzymes associated with reactive oxygen species (ROS) and SOD have been elucidated for heart failure and myocardial cell injury. Mitochondria, the main organelles involved in ROS generation, are the main targets of ROS attacks. The mitochondria-targeted regulation of oxidative stress and antiredox balance and related topics have now gained focus in heart failure research [6, 37].

## 3. Regulation of Cardiotoxic Injury and Mitochondrial Homeostasis in the Pathology of Heart Failure

### 3.1. Mitochondrial Energy Metabolism Dysfunction in Heart Failure

Mitochondrial homeostasis mechanisms are regulated by mitochondrial mass control or monitoring systems [38]. Mitochondria are organelles with phospholipid bilayers and are mainly composed of the mitochondrial outer and inner membranes and mitochondrial matrix [39]. The mitochondrial intermembrane space, located between the outer and inner membranes, plays key roles in transporting proteins across the mitochondrial membrane and in oxidative phosphorylation [39, 40]. The outer mitochondrial membrane has a channel protein that is permeable to small molecules, and the inner mitochondrial membrane increases its surface area through the multifolded mitochondrial cristae structure. The inner membrane assembles channel proteins, almost all electronic respiratory chain complexes, and the ATP enzyme complex [41]. The mitochondrial matrix contains various enzymes and substrates involved in the citric acid cycle. In addition, mitochondria are the only organelles with double-stranded circular DNA capable of relatively independent replication, transcription, and translation [42].

Mitochondria are the power plants for energy production in cardiomyocytes, and the diastolic and systolic activities of the heart consume large amounts of energy [43, 44]. Under normal circumstances, more than 90% of energy can be generated through the mitochondrial oxidative phosphorylation process, and the remaining energy can be generated through the anaerobic glycolysis pathway of fatty acids and glucose [45]. In maintaining normal energy metabolism and the physiological functions of the heart, mitochondria continuously carry out aerobic oxidation processes and generate the energy required by the myocardium [46]. From the perspective of energy supply, most of the energy required by cardiomyocytes comes from fat, and a small part comes from carbohydrates. Free fatty acids and glucose are the main energy-supplying substances and are finally converted into acetyl-CoA in the mitochondria through fatty acid-*β* oxidation and glycolysis. They then participate in the citric acid cycle. Each cycle of fatty acid *β*-oxidation must be catalyzed by different enzymes, including fatty acid-*β* oxidase, long-chain acyl-CoA hydratase, and long-chain 3-ketoacyl-CoA thiolase [47]. The reduced coenzyme I and H^+^ produced in the citric acid cycle can be transported along the electron respiratory chain, enter the mitochondrial matrix through the ATPase complex, release the potential energy in the mitochondrial membrane to catalyze the phosphorylation of adenosine diphosphate, thus, generating ATP for the myocardium. It is required for normal ejection and energy metabolism activities [48].

**Figure 1:**
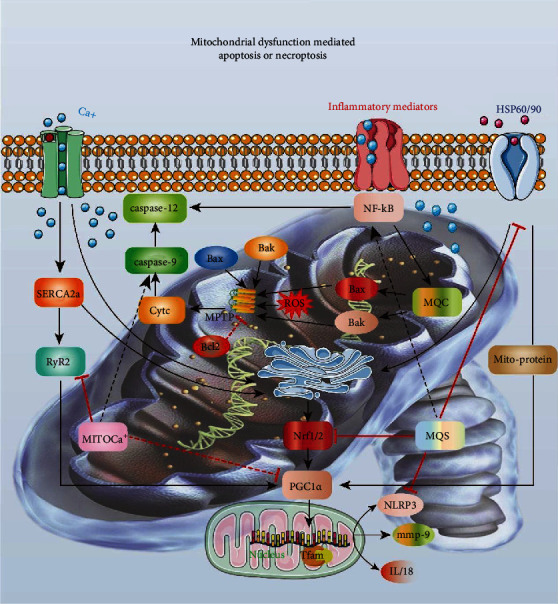
Mitochondrial dysfunction-mediated apoptosis and necroptosis. Oxidative stress injury is the main mechanism of decreased defense function caused by increased mitochondrial ROS generation and decreased activity of antioxidant enzymes such as SOD/GSH/CAT. When myocardial ischemia, hypoxia, and other pathological changes occur, dysfunction in mitochondrial oxidative phosphorylation leads to mitochondrial respiratory chain damage, which directly or indirectly damages myocardial cells. Mitochondrial oxygen free radicals can regulate cellular signal transduction pathways, causing lipid peroxidation, protein function inhibition, and G protein-effector coupling dysregulation, resulting in heart failure. Additionally, the ROS can mediate apoptosis through the caspase pathway. It can inhibit cardiac diastolic and systolic function by modifying myocardial myofibrillar proteins through ROS oxidation, resulting in decreased cardiac function. ROS can also regulate the activity of NF-*κ*B, thereby activating the signaling pathway that induces cardiac hypertrophy and the transcriptional expression of related genes. Mitochondria can also maintain calcium balance in the body by uptake and excretion of Ca^2+^. The concentration of mitochondrial Ca^2+^ plays substantial role in the generation of mitochondrial ATP, the permeability of ion channels, and the regulation of calcium signal transduction pathways. The number of mitochondria is reduced in exhausted cardiomyocytes, and the uptake capacity of the sarcoplasmic reticulum is reduced. Consequently, the cellular accumulation Ca^2+^ becomes excessive, which in turn affects the oxidative phosphorylation process, thereby reducing ATP synthesis and inducing cardiomyocyte apoptosis through the caspase pathway. When calcium overload occurs, excessive cytoplasmic Ca^2+^ is taken up by the mitochondria, the mitochondrial membrane potential decreases, the osmotic transport channels are opened, ATP consumption increases, and myocardial injury occurs. Decreased calcium content in the sarcoplasmic reticulum of cardiomyocytes leads to impaired calcium release and weakened myocardial contractility aggravating the degree of heart failure. Increased Ca^2+^ concentration will activate calcium ion-dependent protein kinases and phospholipases, degrade membrane phospholipids and structural proteins, and increase mitochondrial membrane permeability, causing mitochondrial deformation and swelling. This affects ATP synthesis and aggravates energy metabolism disorders.

As shown in Figure 1, when the oxygen supply to myocardial cells is severely deficient, the cells must rely on the glycolysis pathway for energy production. Experimental studies [49] related to heart failure in rats have found that with the development of heart failure, the ATP generated by substrate oxidation and the utilization of ATP by cardiomyocytes decreases. In the early stages of heart failure, the utilization of myocardial substrates is impaired, the oxidative metabolism of free fatty acids decreases, and the production capacity of glucose oxidative metabolism increases, resulting in decreased ATP production [50, 51]. Therefore, the supply and consumption of energy maintain a balance. In late stages of heart failure, the mitochondrial compensation is weakened, aggravating the metabolic disorder.

In addition, when heart failure has entered the progressive stage, activity of mitochondrial complexes I, III, and IV in cardiomyocytes decreases sharply [25, 52]. This may cause a decrease in ATP synthase activity, affecting the activity of ATP-sensitive potassium channels. The disturbance of ion channel homeostasis may be the main reason for the disturbance of energy metabolism and homeostasis in cardiomyocytes [25, 53]. Mitochondrial biosynthesis disorders begin during the clinical cardiac compensatory hypertrophy stage. In patients with congenital heart disease, defects in mtDNA replication can cause a mitochondrial reduction in the right ventricle, resulting in pathological hypertrophy and heart failure. Mitochondrial energy metabolism disorders caused by cardiomyocyte injury may further affect the development and progression of heart failure, leading to a vicious cycle [54].

### 3.2. Mitochondrial Oxidative Stress and Mitochondrial Dysfunction in Heart Failure

Mitochondria also produce ROS and energy [44]. Excessive accumulation of ROS can lead to the abnormal opening of the mitochondrial permeability transition pore, thereby regulating the permeability of the mitochondrial membrane and causing an imbalance in ion concentrations inside and outside the mitochondria [55]. ROS can activate various transcription factors, such as the Cyt-C transferase, BAX, and caspase pathways. ROS-mediated oxidative stress can also cause mitochondrial structural and functional changes, leading to mitophagy dysfunction [52]. Oxidative stress injury is the pathological process in which the body's ROS production increases and/or the antioxidant defense function decreases. This disrupts the redox balance and causes cellular oxidative stress injury [56, 57].

In myocardial ischemia, dysfunction of mitochondrial oxidative phosphorylation directly or indirectly leads to mitochondrial respiratory chain damage causing myocardial energy transfer disorders [11, 58]. Mitochondrial oxygen free radicals can regulate cellular signal transduction pathways, causing membrane lipid peroxidation, protein function inhibition, and G protein-effector coupling dysregulation, resulting in heart failure [59]. ROS can mediate the production of endothelin through the RAS-RAF-ERK pathway. It can also inhibit the heart's diastolic and systolic function by modifying the myocardium's myofibrillar protein through ROS oxidation. ROS can also regulate the activity of NF-*κ*B and activate signaling pathways that induce cardiac hypertrophy and the transcriptional expression of related genes [60].

### 3.3. Mitophagy in Heart Failure

**Figure 2:**
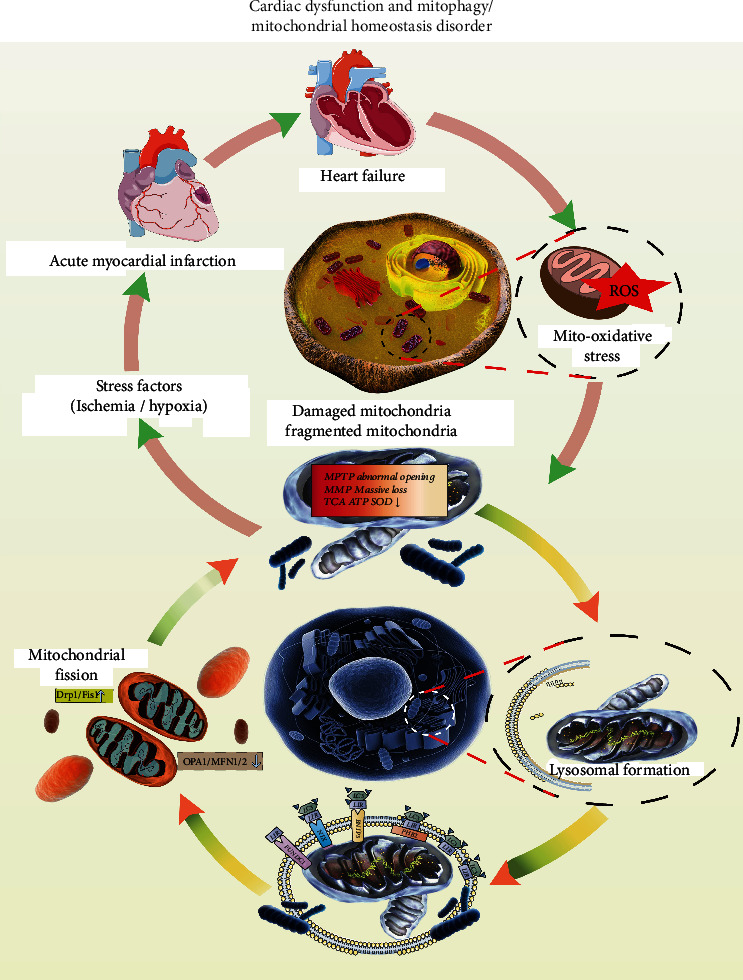
Myocardial injury and mitochondrial homeostasis disorder in heart failure. Mitochondria play an important role in energy production, oxidative stress, maintaining constant intracellular Ca^2+^ concentration, and preserving cellular structural integrity. Moreover, their dysfunction is related to the mechanisms of myocardial injury. Mitophagy can remove damaged mitochondria over time and thus, avoid the ROS-mediated oxidative stress damage and production of certain toxic effects on cells, and effectively maintain homeostasis of the internal cardiomyocyte environment. Cardiomyocyte mitophagy is substantially decreased during the pathological course of heart failure. Impaired mitophagy accelerates the progression of heart failure after myocardial infarction. Damaged mitochondrial DNA induces the mitochondrial fission mechanism, inhibits mitochondrial fusion, and leads to myocardial intracellular calcium overload, inflammatory injury, necrosis, apoptosis, and myocardial fibrosis; it promotes heart failure. The mitochondrial damage mechanism caused by various factors may be one of the pathological mechanisms behind heart failure. The effect of the SOD-led antioxidant defense system is reduced, free radical metabolism is distorted, and oxidative stress levels are enhanced. Mitochondria are the metabolic centers of cellular supply and the preferred targets for cytotoxicity and hypoxia-ischemia damage. Therefore, free radical attack on the mitochondrial membrane may lead to dysregulated mitophagy and excessive mitochondrial fission. This ultimately leads to a “vicious cycle” of enhanced mitochondrial oxidative stress and dysregulation of cellular homeostasis. ROS-mediated oxidative stress is closely associated with mitochondrial damage.

Mitophagy is the body's defense mechanism, the “self-clearing” process of damaged mitochondria within cells. Moreover, during the process of apoptosis or cell death, mitochondrial autophagy and fusion/fission can also interact to jointly maintain cell homeostasis and energy metabolism (Figure 2). External stimuli cause depolarization damage to the mitochondria, and autophagy-related proteins recognize these mitochondria. Under physiological conditions, intracellular mitophagy is maintained at a certain level, removing dysfunctional mitochondria over time and providing raw materials to synthesize fresh mitochondria to promote cell survival [8, 61, 62]. In addition, mitophagy can scavenge the ROS generated during oxidation. Under stress conditions, mitophagy is disturbed, the number of defective mitochondria increase, and a large amount of ROS is produced. Intracellular ROS cannot be cleared in time [63, 64]; it can cause the release of apoptotic factors, induce apoptosis, damage the normal mitochondria, and thus, promote myocardial inflammatory reaction damage and fibrosis. Therefore, increasing the level of mitophagy can promptly remove damaged mitochondria from cells, ensuring the number and function of normal mitochondria [64, 65]. Insufficient or blocked autophagy increases mitochondrial oxidative stress damage and causes heart disease in mice. With increasing age, lysosomes containing lipofuscin granules accumulate excessively in cardiomyocytes. This occupies their effective lysosomal enzymes, which leaves only a tiny portion of the hydrolase for autophagy and other activities, leading to decreased autophagy ability [46]. The experimental results suggest that the progressive reduction of autophagic capacity in aging hearts is partly attributable to lipofuscin accumulation in lysosomes. Peroxidation of the lysosomal membrane may lead to secondary lysosomal rupture, which can release harmful lysosomal enzymes, resulting in programmed cell death or necrosis of cardiomyocytes [66, 67].

Under normal circumstances, excessively elevated or reduced levels of cardiac mitophagy maintain mitochondrial and intracellular protein and organelle metabolism. The level of mitophagy fluctuates according to stress, hypoxia, and nutrient deprivation. Studies have confirmed that insufficient mitophagy leads to myocardial hypertrophy and heart failure, but these studies lack a unified conclusion [68]. Shirakabe et al. [69] proposed that in the progression of heart failure from chronic pressure overload, the mitophagy level is higher than basal during the cardiac compensation and myocardial remodeling protection phases; mitophagy decreases below basal levels during decompensation and myocardial remodeling injury, and heart failure gradually progresses [67]. Transverse aortic coarctation (TAC) was performed in mice to simulate a pathological model of heart failure. The experimental results showed that cardiac hypertrophy occurred five days after the establishment of TAC, accompanied by a decreased mitophagy activity. The ejection fraction decreased 14 days after TAC, and heart failure occurred. Mitophagy was downregulated, followed by severe mitochondrial dysfunction. On day 7 after TAC, injection of a mitophagy inducer indirectly increased the level of mitophagy and alleviated mitochondrial dysfunction in cardiomyocytes. Therefore, mitophagy may be involved in the mechanism underlying myocardial injury associated with heart failure.

## 4. Mitophagy

### 4.1. Mitophagy and Autophagy

Both autophagy and mitophagy are specific autophagic phenomena that selectively remove damaged mitochondria or organelles from within cells. Autophagy and mitophagy are related to a certain extent, and disruptions to their normal functioning are associated with heart failure and myocardial fibrosis. The induction of autophagy can be divided into intracellular (senescent or damaged organelles, misfolded proteins, etc.) and extracellular (starvation, hypoxia, etc.) aspects. Autophagy begins with the establishment of autophagosomes, which involves the expression of a series of autophagy-related genes (ATGs) and the formation of protein complexes [70].

Lysosomes are membranous organelles that break down proteins, nucleic acids, damaged mitochondria, and other biological macromolecules in the cells of protozoa and multicellular animals [71–73]. Mitophagy is a conserved evolutionary process in which cellular proteins and organelles are engulfed by autophagosomes and ultimately delivered to lysosomes for degradation [74]. The formation of the mitophagic lysosome is an important process in the self-repair of cell damage [70]. When the cellular mitochondria are depolarized and damaged, they are specifically encapsulated into autophagosomes and fused with lysosomes for the complete degradation, thus, maintaining intracellular homeostasis [75]. Autophagic lysosomes are vesicles that act on endogenous substrates, i.e., substances resulting from intracellular metamorphosis, injury to certain organelles, or damage to the local cytoplasm [76].

These lysosomes are widely present in normal cells and act as intracellular “scavengers” within a normal pathway for the renewal of mitochondria and other organelles [77]. When various stress stimuli damage tissue cells, autophagy-lysosomes are greatly increased, thereby playing a protective role against cell damage. The substrates for autophagic lysosomes are endogenous—they originate from senescent and disintegrated organelles or the local cytoplasm within the cell [78]. They are enveloped by a single-layer membrane and often contain unbroken endoplasmic reticulum, mitochondria, Golgi complexes, lipids, glycogen, etc. In healthy cells, autophagic lysosomes play an important role in digesting, breaking down, and naturally replacing intracellular structures [79]. When cells are exposed to certain medications, radiation, or mechanical damage, the number of lysosomes increases markedly [80]. Therefore, autophagic lysosomes are often observed in damaged cells. Lysosomes are important centers for intracellular material degradation and signal transduction. Degradation is initiated by various substrates inside and outside the cell, such as fragmented mitochondria and other organelles. Mitochondria and lysosomes are critical to the pathophysiological (mal)functioning of cells. The dysfunction of these organelles is closely associated with cardiomyocyte survival [81].

ULK complexes (including ATG13, ATG101, and FTP200) sense the stimulation of upstream signal nodes and then recruit proteins to form phosphoinositide 3-kinase (PI3K) complexes; PI3K complexes phosphorylate phosphatidylinositol to generate phosphatidylinositol 3-phosphate, which attracts binding proteins as well as ATG2 and ATG9 to the membrane structures. This process induces the formation of the ATG5/12/16 complex, and the light chain 3 (LC3II) of microtubule-associated protein 1 binds to phosphatidylethanolamine on the membrane surface to form LC3II. Autophagosomes are formed at this time; they transport substrates to the lysosome for fusion degradation [82, 83]. Mitophagy mainly induces autophagy through depolarization after mitochondrial damage; the damaged mitochondria and autophagosomes fuse to form mitophagosomes, which then fuse with lysosomes to form mitophagolysosomes; the mitochondria are then degraded within the lysosome [84].

Mitophagy is a major mechanism underlying myocardial fibrosis [85]. Myocardial fibrosis describes the excessive accumulation of extracellular matrix in the myocardial interstitium, an increase in collagen fiber content within the myocardial tissue, an increase in myocardial stiffness, and the close relationship between cardiac structural remodeling, arrhythmia, and heart failure [29]. When myocardial cells are injured by ischemia, hypoxia, or inflammation, they lose many calcium ions and initiate NLRP3-mediated inflammatory responses, eventually leading to myocardial fibrosis [29]. Oxidative stress is involved in this process, and ROS causes myocardial fibrosis by regulating the quantity and quality of the extracellular matrix of the myocardial interstitium [29]. The excessive production of ROS in the mitochondria decreases the antioxidant capacity, damages mitochondrial DNA and protein oxidation functions, promotes myocardial remodeling, and causes myocardial fibrosis. Mitochondrial injury-related molecules are released to the extracellular space after cell injury and act as proinflammatory factors that damage cardiomyocytes. Partially damaged mitochondrial DNA cannot be completely degraded by autophagy, which leads to an inflammatory cardiomyocyte response that induces myocarditis and dilated cardiomyopathy [34]. These myocardial diseases show different degrees of fibrosis and myocardial hypertrophy further aggravating the pathological progression of heart failure [86, 87].

Furthermore, when under hypoxic stress, cardiomyocyte mitochondria produce ROS and other products during energy production [24]. ROS can open the mitochondrial permeation transition pores (mPTP), thereby regulating the permeability of mitochondrial membranes and activating them [88]. Cytochrome C transferase, Bax, caspase, and other transcription factors cause severe cardiomyocyte injury.

Thus, mitophagic regulation plays a role in maintaining the internal balance of myocardial cells and delaying the process of myocardial fibrosis, which is a key problem that needs to be resolved urgently.

### 4.2. FUNDC1-Mediated Receptor-Dependent Mitophagy in Heart Failure

**Figure 3:**
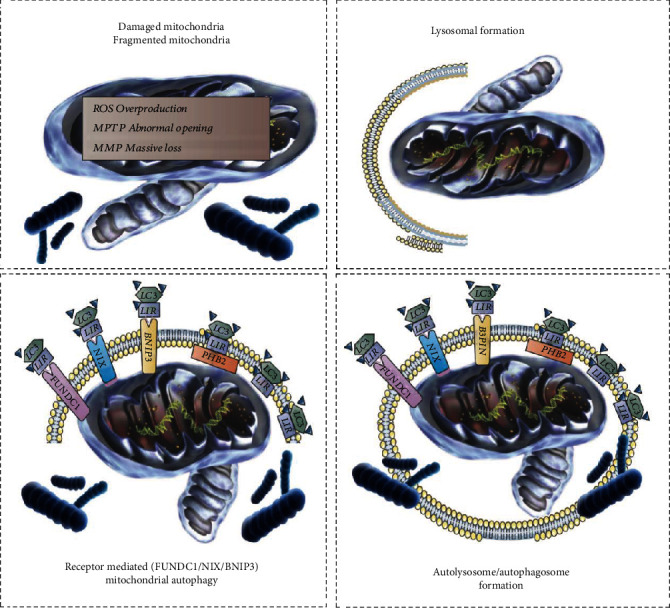
Regulatory mechanisms of receptor-dependent mitophagy mediated by BNIP3 and FUNDC1 in cellular physiology and pathology. BNIP3 and NIX are multifunctional mitochondrial outer membrane proteins. In myocardial tissue, BNIP3 and NIX expression are regulated by hypoxia, and promote cardiomyocyte apoptosis, resulting in decreased cardiac function. However, BNIP3 and NIX localized to the mitochondrial outer membrane can also act as mitophagy receptors, by binding LC3 through their LC3 interacting regions (LIRs), and directly recruit phagocytic vesicles to the mitochondria to mediate mitophagy. Oxidative stress induces BNIP3 homodimerization to activate mitophagy. Furthermore, BNIP3 and NIX may directly activate mitophagy by disrupting the interaction of BcL-2 with Beclin1. FUNDC1 is a highly conserved mitochondrial outer membrane protein that is widely expressed in various cells, tissues, and the heart. As a mitophagy receptor, it mediates mitophagy by interacting with LC3. Under stress conditions such as hypoxia and mitochondrial membrane uncoupler treatment, FUNDC1 is activated by phosphorylation of its serine 17 site by ULK1 and dephosphorylation of its serine 13 site by PGAM5, enhancing the binding of its LIR to LC3 to promote mitophagy. The mitophagy receptor is PHB2. Although PHB2 has LIR in the inner mitochondrial membrane, LC3 usually cannot reach it. Therefore, its mediated mitophagy relies on the outer mitochondrial membrane (OMM) protein degradation mechanism that can lead to the rupture of the OMM. In mammalian cells, Parkin-mediated mitophagy also requires the regulation of PHB2, especially in response to oligomycin-induced mitochondrial stress, but its association with other stressors remains to be further verified.

Based on the different regulatory mechanisms and pathways, mitophagy can be divided into receptor-mediated and nonreceptor-mediated mitophagy [62]. There are some special proteins and lipids in the mitochondrial outer membrane (including FUNDC1/BNIP3 and NIX), and these different protein types can directly act as mitophagy receptors [63] (Figure 3). Therefore, ubiquitination and adaptor proteins are not required to complete mitophagy.

Mitochondrial cardiolipin and receptor protein FUN14 or inclusion (FUNDC1) can directly bind to LC3 on autophagosomes and exert this effect [89, 90]. FUNDC1, a mitophagy receptor, also has an LIR. Hypoxia can induce dephosphorylation of FUNDC1, enhance the interaction between FUNDC1 and LC3, and promote mitophagy. B lymphocytoma-2 (Bcl-2)/adenovirus E1B 19 kDa interacting protein 3 (BNIP3) and NIX/BNIP3L are proapoptotic receptor proteins located on the outer mitochondrial membrane [91].

Although all these indicators can induce mitochondrial dysfunction and inhibition of the interaction between BNIP3 and LC3 can substantially affect activation of mitochondrial autophagy, mitochondrial autophagy is still not completely inhibited. This offers additional evidence for the existence of other autophagy receptors on the inner or outer mitochondrial membrane, which may be related to FUNDC1-mediated receptor-dependent mitochondrial autophagy [92]. Studies have shown that receptor-mediated mitophagy is important for maintaining a steady mitochondrial state under normal conditions. Conversely, under the influence of pressure, the PINK1/Parkin pathway preferentially clears dysfunctional mitochondria [93]. Although these functions may appear independent, there may be substantial interconnections between the two pathways. However, it is important to note that mitophagy must maintain a degree of balance. Under normal conditions, intracellular environmental or mitochondrial homeostasis mechanisms require a certain level of autophagy to regulate cellular organelles and proteins. Excessive autophagy may lead to an imbalance in the cellular energy metabolism levels and activation of the apoptosis pathway because the proteins and organelles necessary for ATP production or mitochondrial respiratory function are also excessively cleared, and thus, the supply and demand of ATP are imbalanced. This may be one of the main causes of cell death [94]. Excessive autophagy is specifically associated with cardiomyocyte death.

Therapeutic approaches targeting mitophagy represent a promising strategy for repairing damaged myocardium [9, 12]. Mitophagy is rapidly induced after adult cardiac progenitor cell (CPC) differentiation [95, 96]. Conversely, mitophagy promotes an appropriate mitochondrial network in CPCs during differentiation. Knockdown of BNIP3 and FUNDC1 during differentiation results in sustained mitochondrial fission and fragmentation. It can also lead to increased cell death and the nonviability of infarcted hearts [97].

ALDH2 has produced certain activities of nitrate tolerance and diabetes in animal models [98]. The antioxidant *α*-lipoic acid (*α*-LA) exerts a regulatory role in heart failure and mitochondrial injury through FUNDC1. During the assessment of myocardial function and mitophagy in ALDH2 knockout (ALDH2-/-) mice, *α*-LA acted to restore the activity and expression of ALDH2, increase the expression level of the mitophagy receptor protein FUNDC1, and reduce TAC-induced ventricular hypertrophy and ventricular dysfunction. Furthermore, increased ALDH2 activity activated by *α*-LA controlled activation of the Nrf1-FUNDC1 cascade.

Another study [99] found that FUNDC1 deficiency affects high-fat diet- (HFD-) induced cardiac abnormalities. Following depletion of FUNDC1, rats developed metabolic disturbances, marked cardiac remodeling, contractility, and intracellular Ca^+^ and mitochondrial abnormalities upon introducing HFD. FUNDC1 ablation can also inhibit mitophagy and indirectly affect the HFD-induced elevation of fatty acid synthase ACSL4, necroptosis, inflammation, ferroptosis, production of mitochondrial ROS, and induced mitochondrial damage. Without FUNDC1, cytochrome C release, cardiomyocyte defects, and increased lipid peroxidation levels also occur. The chain reaction caused by FUNDC1 deficiency can be interrupted by the inhibitors SP1, ACSL4, and ferroptosis. Experimental data suggest that the pathological mechanisms of cardiac remodeling and dysfunction are closely associated with FUNDC1-mediated mitophagy and ACSL4-mediated ferroptosis.

A study targeting FUNDC1 [100] found that FUNDC1-mediated mitophagy can maintain mitochondrial homeostasis and exert a certain level of protection in reperfused cardiac tissue. Mst1 can regulate levels of chronic cardiometabolic injury and mitophagy; it is also involved in cardiomyocyte death by regulating FUNDC1-related mitophagy. Experimental data suggest that Mst1 substantially increases in the myocardium after reperfusion injury. However, Mst1-KO mice showed a marked reduction in the myocardial infarction area, increased FUNDC1 expression, and increased mitophagy levels. Cardiac ejection function was maintained and the level of myocardial cell death due to ischemia and hypoxia was reduced. Activation of Mst1 inhibited FUNDC1 expression, further inhibiting mitophagy and promoting ROS production. The mitochondrial membrane potential was greatly reduced, and the caspase-9-related apoptotic pathways were activated in cardiomyocytes. Deleting Mst1 reversed FUNDC1 expression in cellular experiments, thereby reactivating and protecting mitophagy and effectively maintaining mitochondrial homeostasis after hypoxia.

Molecular biological studies have also demonstrated that Mst1 can regulate the expression of FUNDC1 through the MAPK/ERK-CREB pathway. These data demonstrate that Mst1 deficiency reduces cardiomyocyte mitochondrial apoptosis by reversing FUNDC1-related mitophagy, thereby sending a prosurvival signal to the reperfused heart. This suggests that Mst1 acts as a novel regulator of cardiac reperfusion injury. The research above discusses the regulatory role of FUNDC1-mediated receptor-dependent autophagy in the pathology of heart failure and provides a good reference for future studies targeting mitophagy in heart failure. However, in addition to the FUNDC1-mediated receptor-dependent autophagy, the PINK1/Parkin pathway-mediated receptor-independent mitophagy in heart failure deserves further attention.

### 4.3. PINK/Parkin-Mediated Receptor-Independent Mitophagy in Heart Failure

**Figure 4:**
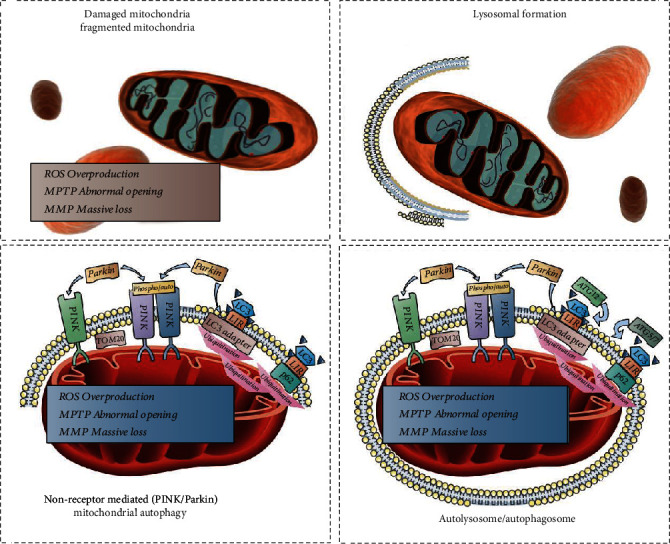
Regulatory mechanism of PINK/Parkin-mediated receptor-independent mitophagy during cell injury. Under the stimulation of inflammation/high glucose/high-fat conditions, excessive ROS will be generated. The permeability transition pore of the mitochondrial membrane will be excessively porous, the MMP (ΔΨ*m*) will be severely reduced, and mitochondrial autophagy will disperse. Enzyme formation: In this abnormal state or in mitochondrial damage, activated PINK will recruit Parkin to the damaged mitochondria. Parkin will further mediate the ubiquitination of mitochondrial outer membrane proteins and promote the recognition of ubiquitinated mitochondria by adaptor proteins. Adaptor proteins link ubiquitin on the mitochondria to LC3 and LIR on the mitochondrial phagosomes, which eventually phagocytose and degrade the mitochondria.

The PINK1/Parkin pathway is important for non-receptor-mediated mitophagy [101]. In healthy cells, PINK1 can translocate into the mitochondria, where it is then actively degraded by mitochondrial processing peptidase/protease (MPP) [102].  Figure 4 shows that when mitochondria are stressed or damaged, there is a substantial loss of mitochondrial membrane potential (MMP). At this time, PINK1 no longer translocates to the inner mitochondrial membrane, instead, accumulates on the outer mitochondrial membrane [103]. PINK1 can be activated by phosphorylation and ubiquitinated on serine 65 (Ser65) while recruiting Parkin. On the mitochondrial membrane, Parkin can also lead to polyubiquitination of its substrate [104]. The ubiquitination of several receptor proteins on the mitochondrial outer membrane, such as hexokinase I, voltage-dependent anion channel 1 (VDAC1), the mitochondrial fusion protein 1/2 (Mfn1/2), and mitochondrial adaptor protein Miro, is aggregated by Parkin activation. In addition, mitophagy mediated by the PINK1/Parkin pathway is closely associated with mitochondrial dynamics mediated by the mitochondrial fusion protein Mfn1/2 [105]. The degradation of the fusion protein Mfn1/2 up to a certain level can maintain the mitochondria in a fission state, which may be necessary for mitophagy. Increased levels of mitophagy protect cardiomyocytes under certain stress conditions [106]. For example, Parkin-mediated mitophagy protects against high glucose- and palmitate-induced cardiomyocyte injury.

Acetylcholine promotes the translocation of PINK1/Parkin to the mitochondria to stimulate protective mitophagy in cardiomyocytes. This may provide a beneficial target for maintaining hypoxia/reoxygenation-injured cardiomyocyte homeostasis [102]. Another study found that the recovery of myocardial contractility in Parkin-deficient mice was blocked after sepsis, and the mitophagy process was also blocked. The Parkin-deficient mice had worse survival than wild-type mice after myocardial infarction. Infarct size increased [103]. Further, the effect of PINK1-deficient cardiac ischemia/reperfusion injury on myocardial injury is exacerbated further than in the wild-type, and overexpression of PINK1 in HL-1 cardiac cells protects cardiomyocytes from hypoxia/reoxygenation injury [107].

Wang et al. determined the role of AMP-activated protein kinase (AMPK) in mitophagy during heart failure [108]. An isoform switch from AMPK*α*2/AMPK*α*1 was observed in samples from mice and patients with heart failure. This switch was also associated with decreased mitophagy and mitochondrial dysfunction. AMPK*α*2-/- mutant mice exhibited suppressed levels of cardiac mitophagy, which worsened the early progression of transverse aortic constriction-induced heart failure. The knockdown of AMPK*α*2 further inhibited the phenylephrine-induced compensatory increase in mitophagy. AMPK*α*2 specifically interacts with phosphorylated PINK1 (PTEN-induced putative kinase 1) at Ser495 with phenylephrine stimulation. The mitophagy increase is accompanied by the elimination of damaged mitochondria, reduced ROS production, and decreased apoptosis in cardiomyocytes. However, the Ala mutation in PINK1 can partially inhibit AMPK*α*2-induced mitophagy. Moreover, in heart failure, the major AMPK*α* isoform can be converted from AMPK*α*2 to AMPK*α*1, accelerating the heart failure pathology. Ran et al. further reported that Samm50, a key positive regulator of cardiac hypertrophy, was downregulated in both pressure overload-induced hypertrophic heart and angiotensin II-induced cardiomyocyte hypertrophy. Samm50 significantly inhibited angiotensin II-induced autophagy activation, as manifested by decreased mitophagy protein levels; however, the inhibition of mitophagy by Vps34 inhibitor or PINK1 knockdown abolished the protective effect of Samm50 deficiency on cardiac hypertrophy. Further research has shown that the protein interaction mechanism of Samm50 and Pink1 can stimulate the accumulation of Parkin in mitochondria to initiate mitophagy.

Zhu et al. found that receptor-interacting protein 3- (RIPK3-) mediated necroptosis plays a key role in cardiac remodeling [109]. The expression level of RIPK3 was further upregulated in myocardial tissue after myocardial infarction in a mouse model of coronary artery ligation and in myocardial cells after hypoxic injury in vitro. Increased RIPK3 expression is also associated with severe cardiac remodeling, cardiac insufficiency, and higher mortality. This suggests that RIPK3 is an important regulatory protein that mediates the pathological mechanisms of heart failure. RIPK3 overexpression subsequently suppressed AMPK- and Parkin-mediated mitophagy. Decreased levels of mitophagy further increase the opening of the mPTP, ultimately leading to increased levels of cardiomyocyte necroptosis. Simultaneously, knockdown of the RIPK3 gene can induce AMPK/Parkin-mediated mitophagy activation, which ultimately inhibits mPTP opening, alleviates cardiac remodeling after myocardial infarction (MI), and inhibits cardiomyocyte necroptosis.

### 4.4. Regulatory Mechanism and Function of Transcription Factor EB in Mitochondrial Autophagy

Transcription factor EB (TFEB), a member of the MiTF/TFE family of leucine zipper transcription factors, plays an important role in the regulation of autophagy [110]. Transcription of TFEB can activate the autophagy pathway; induce the expression of key genes in the stages of autophagic vesicle formation, autophagic vesicle extension, content recognition, and content degradation; and control the activation of autophagy/mitophagy [111]. TFEB recognizes E-box and M-box promoters to initiate the transcription of corresponding genes, and it regulates the biological process of cellular energy metabolism and the pathological process of cell damage [112]. Moreover, a study found that TFEB can protect against myocardial or cardiomyocyte injury by activating the autophagy-lysosomal pathway.

**Figure 5:**
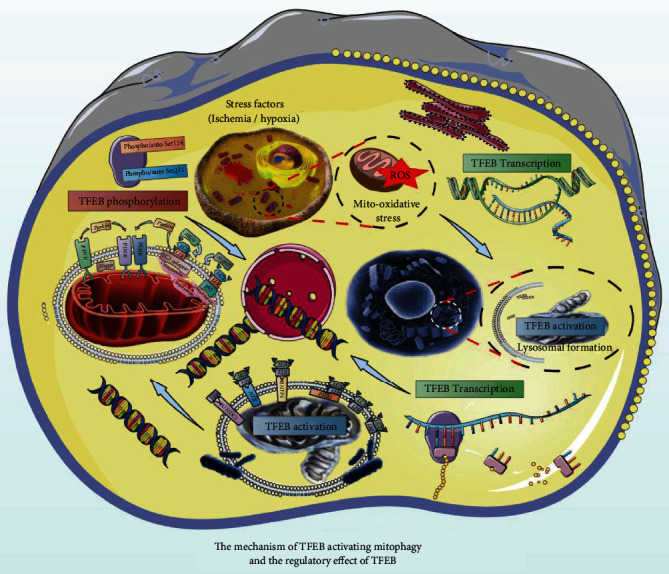
Regulatory mechanism and function of TFEB in mitochondrial autophagy. TFEB is an important transcription factor in cells and plays an essential role in the lysosome and autophagy-related processes. The expression of various autophagic genes and the synthesis of lysosomes are associated with TFEB and play an important role in the occurrence and development of various diseases. The formation of autophagolysosomes is regulated by the phosphorylation of TFEB, which is mainly located in the cytoplasm. When cardiomyocytes are under hypoxic or ischemic stress, ROS generation can lead to the activation of TFEB and the activation of transcriptional levels, the formation of autophagy-lysosomes, and the regulation of TFEB phosphorylation and transcription can also affect mitochondrial autophagy. The regulation of phagocytosis maintains the homeostasis of the intracellular environment under certain conditions.

Figure 5 shows that TFEB plays a role in regulating cellular energy metabolism and homeostasis of the intracellular environment. It generally exists in the cytoplasm and can enter the nucleus under stress to further initiate the transcription of target genes. Under stress conditions or impairment of lysosomal function, upregulation of TFEB expression can increase the number of autophagosomes, promote lysosomal turnover, and increase autophagic flux [113]. TFEB is closely associated with lysosome synthesis, impaired autophagic degradation, poor fusion of autophagosomes and lysosomes, and lysosomal dysfunction. The regulation mechanisms by TFEB and lysosomes may be central to autophagy regulation [114]. Lysosomal biosynthesis is tightly controlled by the cellular metabolic state and by signaling from lysosomal membrane-bound molecules. As the master switch of lysosome generation and autophagy, TFEB coordinates cellular responses to various stresses, such as starvation, metabolic stress, and lysosomal stress [115, 116].

In total, more than 400 TFEB target genes have been identified, many of which are directly associated with the activation of lysosomes and autophagy. These include lysosomal hydrolase and accessory protein genes, lysosomal membrane protein base domains, lysosomal V-ATPase pump genes, and lysosomal biosynthesis regulator genes [110]. Lysosomes are organelles with digestive and transport functions that regulate calcium channels, and calcium ion imbalance may disrupt lysosome function. TFEB activates the transcription of genes encoding proteins involved in many aspects of cellular clearance, such as lysosomal biogenesis, autophagy, exocytosis, endocytosis, and other lysosome-related processes [117, 118].

TFEB Ser142 and Ser211 are hyperphosphorylated in the cytoplasm, and under stress conditions such as starvation or lysosomal dysfunction, TFEB is rapidly translocated to the nucleus [111]. This process is regulated by the phosphorylation of TFEB, which is mainly located in the cytoplasm, while the dephosphorylated form is generally found in the nucleus. TFEB is involved in the lysosome-to-nucleus signaling mechanism, transmitting information about the state of the lysosome to the nucleus to trigger transcriptional reactions, which further influence the process of crosstalk between lysosomes and the nucleus, controlling cellular clearance and energy metabolism [119, 120].

### 4.5. Regulation of Mitophagy in Apoptosis Induced by Cytotoxicity

**Figure 6:**
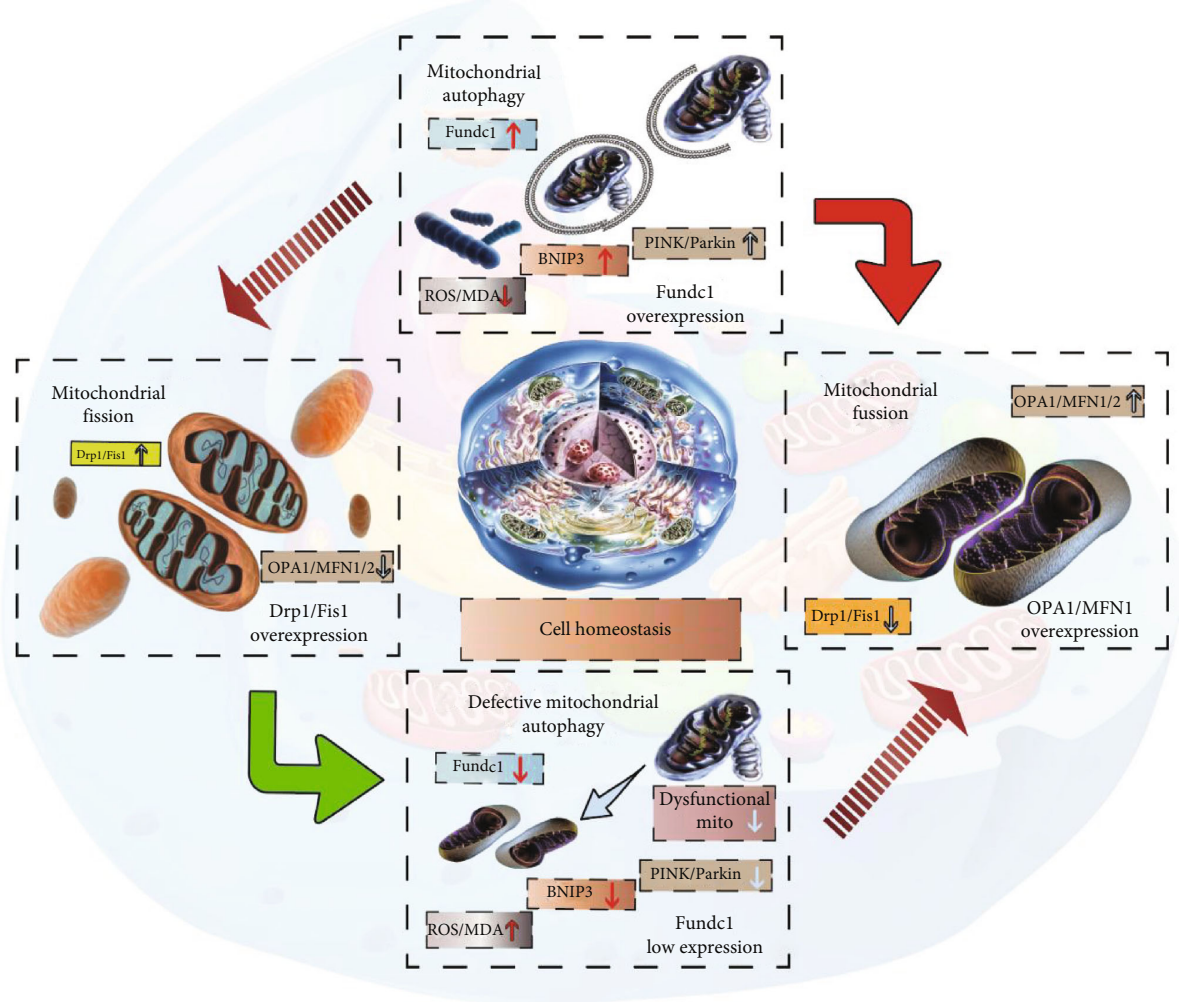
Mitochondrial autophagy dominated mitochondrial quality control and intracellular environmental homeostasis. Interaction of receptor-mediated and non-receptor-mediated mitophagy with cellular homeostasis. In conditions causing mitochondrial stress, the mitochondrial fission kinesins Drp1 and Fis1 mediated mitochondrial fission. Additionally, the expression of Opa1 and Mfn1/2 decreased, the Opa1- and Mfn1/2-mediated mitochondrial fusion decreased, and the mitochondrial fragmentation increased the level of chemistry. Elevated levels of mitochondrial fragmentation led to insufficient mitochondrial ATP production, increased dysfunctional and incomplete mitochondria, activation of receptor-dependent mitophagy mediated by FUNDC1 and BNIP3, and activation of PINK/Parkin-mediated nonreceptor mitochondrial autophagy. Body-dependent mitophagy levels were also activated; however, neither autophagy pathway could completely eliminate or digest the incomplete mitochondrial debris. The intervention of mitophagy-targeted drugs or activators/inhibitors led to inhibition of Drp1 and Fis1 and increases of Opa1 and Mfn1/2 expression. Mitochondrial fission was also excessively inhibited, and mitochondrial fusion was activated. In a balanced state of mitochondrial fusion and fission, the level of mitochondrial fragmentation decreases. The receptor-dependent mitophagy mediated by FUNDC1 and BNIP3 and receptor-independent mitophagy mediated by PINK/Parkin can clear or degrade mitochondria, maintain mitochondrial and cellular homeostasis, and protect cardiomyocytes. Mitophagy is transiently activated in mouse myocardial injury and downregulated in response to stress overload. Mitophagy plays key roles in mediating mitochondrial dysfunction and heart failure development. Restoration of mitophagy alleviates cardiac dysfunction during stress overload. During cardiac decompensation, pathological remodeling of the myocardium is associated with Drp1-mediated mitophagy. An insufficient level of mitophagy also accelerates the process of decompensation and promotes heart failure progression. This may be closely related to mitochondrial fusion/fission.

Notably, mitophagy plays a bidirectional regulatory role in the protection of cardiomyocytes [121]. The abovementioned studies introduced the effects of FUNDC1-mediated receptor-dependent mitophagy and PINK/Parkin-mediated receptor-independent mitophagy on the pathological mechanism of heart failure [122]. The increase in mitophagy could have a certain protective effect on cardiomyocytes and simultaneously inhibit the level of apoptosis [122].  Figure 6 shows that mitochondrial autophagy has a bidirectional regulatory effect on intracellular homeostasis, and studies have found that inhibiting excessive mitochondrial autophagy can protect cardiomyocytes and improve cardiac function [122, 123]. Peng et al. [124] found that PINK1/Parkin-mediated mitochondria played a central regulatory role in DOX-induced cardiotoxicity. Experimental evidence suggests that PINK1/Parkin is a key pathway that regulates mitophagy [125]. PINK1/Parkin-mediated overactivation of mitophagy are key factors contributing to DOX-induced cardiac injury [124, 126]. This was demonstrated by DOX-induced increases in the mitophagosome markers LC3 and Beclin1, which were decreased by p62. DOX can activate PINK1/Parkin and promote its translocation to the mitochondria [127]. However, the mitochondrial fission inhibitor mdivi-1 can inhibit mitophagy's activation and maintain the mitochondrial biosynthesis level, thereby alleviating mitochondrial dysfunction [128]. Consistent with this result, Mito-tempo could also scavenge mitochondrial superoxide, attenuate the activation of PINK1/Parkin, and protect cells from DOX-induced adverse effects. It can be inferred that the PINK1/Parkin pathway plays an important regulatory role in DOX-induced cardiotoxicity injury, and inhibition of PINK1/Parkin can improve DOX-induced cardiotoxicity injury [129]. The experimental data further verified the bidirectional regulation of mitochondrial autophagy, suggesting that it may be the key condition for maintaining normal mitochondrial function.

Zhou et al. [130] also found that melatonin could improve cardiac function, restore blood flow, and reduce microcirculatory defects during the treatment of microvascular ischemia/reperfusion injury. Cardiac microcirculatory endothelial cells (CMECs) from melatonin-treated mice had an intact endothelial barrier and increased endothelial nitric oxide synthase expression. Notably, AMPK*α* deficiency nullified the beneficial effects of melatonin on the microvasculature. PINK1/Parkin is upregulated and mediates mitophagy, ultimately leading to the death of coronary microvascular CMEC. However, melatonin inhibits mitochondrial fission, restores VDAC1-glucokinase 2 interaction, prevents mPTP opening and PINK1/Parkin activation, and ultimately blocks PINK1/Parkin-mediated mitogenesis. The above studies have explained the different mechanisms of mitophagy in heart failure, myocardial hypoxia, ischemia/reperfusion injury, and the FUNDC1-mediated receptor-dependent mechanism in different stages of myocardial ischemia and reperfusion. The changes in PINK/Parkin-mediated receptor-independent mitophagy and the changes in mitophagy within the pathology of restricted and unrestricted heart failure also deserve further discussion and research. Therefore, the double-edged sword of mitophagy needs to be used to conceive of and develop new pharmacological approaches to alleviate MI-related ischemic stress and MI, with consideration of the different stages of myocardial injury. This will involve various administration times and dosages, but it is important that a series of targeted studies on mitophagy be conducted in the future.

## 5. Natural Medicinal Plants and Active Ingredients in the Regulatory Mechanism of Mitophagy in Heart Failure

**Figure 7:**
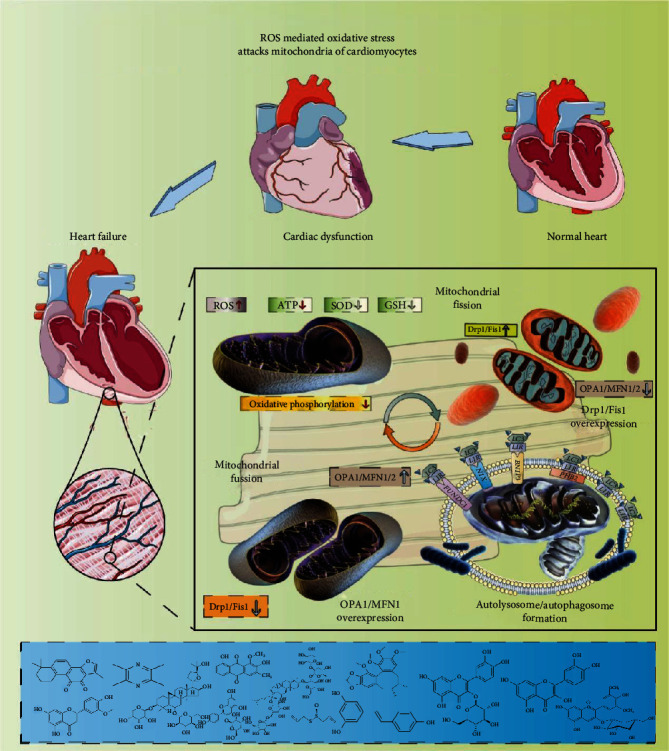
Regulatory mechanism of natural medicinal plants in maintaining mitochondrial homeostasis by regulating oxidative stress and mitophagy, mitochondrial fusion/fission (receptor-mediated/non-receptor-mediated). Oxidative stress refers to a relatively unbalanced state with excessive ROS production or insufficient antioxidant capacity in the body. The activity of SOD/GSH is insufficient, the production of ATP is inadequate, and oxidative phosphorylation is decreased. Substances like superoxide radicals, hydrogen peroxide, and hydroxyl radicals are collectively referred to as “ROS.” Under physiological conditions, mitochondria generate ATP through oxidative phosphorylation to provide energy for cells, and the electron reduction process in the respiratory chain reduces a small amount of oxygen to ROS. However, increased production of ROS and insufficient antioxidant capacity result in ROS accumulation. Mitophagy and mitochondrial fusion/fission work synergistically and interactively to suppress oxidative stress injury and maintain cellular homeostasis during mitochondrial oxidative stress injury. However, insufficient mitophagy activation and increased mitochondrial fission levels can also lead to mitochondrial dysfunction when there is excessive oxidative stress. Natural medicinal plants can further affect mitochondrial redox balance and maintain mitochondrial functions, including ATP generation and activation of oxidative phosphorylation.

The current treatment methods for heart failure are mainly focused on reducing the workload of the failing heart and striving to achieve a certain level of energy balance of supply and demand. However, maintaining the energy supply inside cardiomyocytes from the mitochondrial perspective is a key issue worthy of study [131, 132]. Mitophagy can be targeted in different stages of heart failure. Mitophagy activation can inhibit cardiac hypertrophy, regulate protein quality, and reduce protein aggregation and the cytotoxicity caused by misfolded proteins [133]. Mitophagy activation can also inhibit the development and progression of heart failure. It can be speculated that coordinated enhancement of autophagosome formation and regulation of autophagosome-lysosome fusion and degradation can restore or enhance autophagic flux and prevent or alleviate the development of the pathological myocardial remodeling that occurs in the decompensated stages [134]. Inhibiting the development or progression of cardiac hypertrophy and regulating the quality of intracellular proteins can improve mitophagy and prevent or treat heart failure [135] (Figure 7). However, excessive autophagy damages important organelles and proteins, affecting myocardial energy metabolism. Therefore, in-depth research on the regulatory mechanisms of mitophagy in different states can provide new ideas for the prevention and treatment of heart failure.

### 5.1. Natural Medicinal Plants Protect Cardiomyocytes by Regulating Mitophagy

Natural medicinal plants contain various chemical components, and studies have found that alkaloids, polysaccharides, glycosides, flavonoids, and enzymes are all active compounds with therapeutic potential [9, 13]. Natural medicines and their active compounds have multitarget comprehensive curative effects [136, 137] (Figure 7). Many natural medicines and their active compounds, such as aconite, bupleurum, and astragalus, protect cardiomyocytes, enhance myocardial contractility, dilate blood vessels, induce diuresis, and reduce ventricular remodeling; these are widely used in the treatment of heart failure [12, 106]. Studies have also found that natural active compounds extracted from traditional natural medicinal plants have anti-inflammatory, antioxidative, and antiapoptotic actions, while improving fatty acid oxidation and autophagic effects [130, 136].

#### 5.1.1. Ophiopogon D

The natural active components of steroidal saponins extracted from Ophiopogon japonicus can reduce MMP damage through active antioxidant components, improve mitochondrial energy dysfunction, and balance myocardial energy homeostasis. It can also regulate mitochondrial autophagy, exerting a comprehensive myocardial protective effect [138]. In another study [139], Ophiopogon D (OP-D) promoted antioxidant protection of the cardiovascular system and cardiomyocytes [140]. DOX induced excessive autophagy by generating ROS in H9C2 cells and the myocardium, with a substantial increase in the number of autophagic vacuoles and upregulation of LC3-II/I expression. OP-D treatment significantly alleviated the MMP disruption. It reduced ROS production from mitochondrial damage, thereby inhibiting autophagic activity. This offers some explanation of its protective effect against DOX-induced cardiac toxicity.

#### 5.1.2. Catalpol

Catalpol, a component of Rehmannia glutinosa, has been widely used to protect cardiomyocytes from myocardial ischemia [141]. Catalpol has been shown to exert a protective effect on a heart with glucose-starved H9C2 cells, with antiapoptotic and antioxidant effects. Catalpol protected H9C2 cells from glucose starvation by reducing apoptosis and attenuating oxidative damage. The experimental results also showed that autophagy-related proteins were significantly increased in catalpol-treated cells, indicating that catalpol could further upregulate autophagy. Notably, the autophagy inhibitor (3-MA) eliminated the antiapoptotic and antioxidant effects of catalpol on cardiomyocytes and prevented catalpol-induced mitophagy. The study also found that the estrogen receptor inhibitor, tamoxifen, nullified the activity of mitochondrial-related proteins (LC3, Beclin 1, p62, and ATG5) activated by catalpol. Collectively, these data suggest that catalpol inhibits apoptosis and oxidative stress in glucose-deprived H9C2 cells by promoting mitophagy [142].

#### 5.1.3. Ferulic Acid

Ferulic acid is the main active organic acid component of the classic natural medicine Chuanxiong [143]. In Chuanxiong, ferulic acid exists in both bound and free forms. Therefore, the total ferulic acid content is usually used as an index for the quality evaluation of Chuanxiong. The study found that ferulic acid can increase the level of autophagy through PI3K/Akt/mTOR in a dose-dependent manner. It also improves myocardial injury, regulates serum lactate dehydrogenase (LDH), creatine kinase (CK), and cardiac troponin levels, and improves the injured myocardium's histological characteristics. The cardioprotective mechanism of organic acids may be directly related to activating signaling pathways and restoring autophagic flux [144].

#### 5.1.4. Asiatic Acid

Disturbed cardiomyocyte energy metabolism is a major factor in the pathology of heart failure, which may ultimately lead to a poor prognosis [145]. Mitophagy is important in regulating the mitochondrial respiratory chain function and ATP production. Asiatic acid (AA) is a pentacyclic triterpenoid derived from the traditional natural medicine, Centella asiatica, with anti-inflammatory, antioxidant, and antiapoptotic activities. AA has been found to improve the balance of energy metabolism in cardiomyocytes by activating mitophagy, thereby exerting a cardioprotective effect [146]. Treatment with different doses of AA increased cell viability, ATP levels, and the phosphocreatine/ATP ratio and improved cardiomyocyte energy metabolism. In vivo mouse model studies have found that AA can play a cardioprotective role. AA can promote mitophagy and alleviate dysregulation of mitochondrial homeostasis, which can increase the expression of LC3-II through the number of mitophagosomes in ischemic myocardium in vivo.

AMPK knockdown abolished the mitophagic regulatory effects of AA. Furthermore, PI3K or Akt inhibitors blocked the AA effects on glycophagocytosis/glycolysis and mitochondrial energy metabolism. Although the mechanism by which these natural medicinal plants regulate mitophagy to protect cardiomyocytes has been verified, the specifically targeted regulation mechanism of mitophagy has not been elucidated. Therefore, further recovery experiments are needed to verify whether autophagy regulation is mediated by receptors (i.e., whether it is through the receptor-mediated or non-receptor-mediated pathways).

### 5.2. Natural Medicinal Plants Protect Cardiomyocytes through PINK/Parkin-Mediated Receptor-Independent Mitophagy

Natural medicinal plants can improve myocardial injury and treat heart failure by regulating the mitophagy pathway [64]. Currently, mitophagy regulation by traditional Chinese medicine is concentrated mainly on the PINK1/Parkin and FUNDC1 pathways, and the role of other regulatory pathways is unclear. Chinese medicine has provided new ideas for the prevention and treatment of heart failure. Many recent studies have shown that natural medicinal plants can protect cardiomyocytes by regulating mitochondrial oxidative stress and mitochondrial homeostasis (Figure 7).

#### 5.2.1. Salvianolic Acid B

Salvianolic acid B (Sal B) is a major active component of the natural medicine Salvia miltiorrhiza [64, 147, 148]. Sal B can play a role in the models of isoproterenol- (ISO-) induced myocardial ischemia and lipopolysaccharide + adenosine triphosphate-induced H9C2 cell inflammation. Different concentrations of Sal B have been shown to reduce acute myocardial injury. In vitro studies have also found that expression of PINK/Parkin-mediated mitophagy decreased after modeling, with excessive ROS accumulation and increased mitochondrial membrane potential. Sal B treatment can inhibit intracellular ROS generation, which in turn increases MMP, regulates the expression of PINK/Parkin-mediated mitophagy-related proteins, and inhibits the NLRP3 inflammasome and apoptosis [149].

#### 5.2.2. Resveratrol

Resveratrol is an important antioxidant component of various natural drugs and plays a protective role for cardiomyocytes and vascular endothelial cells in different experimental models (inflammation/hypoxia/ischemia) [11, 150]. Resveratrol can correct aging-induced abnormal mitochondrial morphology and autophagy disorders in H9C2 cells [151]. The inhibition of Drp1 expression significantly increased mitochondrial length. However, aging-mimic cardiomyocytes were resistant to mitochondrial depolarization, showed suppressed expression of PINK1/Parkin and LC3-II and inhibited mitochondrial autophagy. Resveratrol intervention increased Drp1 expression, improved mitochondrial length, and increased mitochondrial translocation of PINK1/Parkin. The activation of PINK1/Parkin may be a potential mechanism by which resveratrol improves cardiomyocyte injury.

#### 5.2.3. Berberine

Berberine (BBR) protects cardiac function in heart failure [57, 152]. The study found various beneficial effects of BBR on chronic heart failure. TAC-induced pressure overload led to cardiac hypertrophy, fibrosis, and cardiomyocyte mitochondrial damage with a concomitant decrease in mitophagy levels, and BBR attenuated these effects [153]. PINK1/Parkin was significantly downregulated in TAC-induced heart failure, while BBR upregulated PINK1/Parkin-mediated mitophagy. Notably, PINK1 knockdown significantly inhibited Parkin-mediated mitochondrial ubiquitination and counteracted the beneficial effects of BBR on heart failure. These data suggest that BBR plays a key regulatory role in reducing cardiac function during cardiac hypertrophy and heart failure. Therefore, we infer that BBR activates mitophagy mainly through the PINK1/Parkin/ubiquitination pathway.

#### 5.2.4. Rosmarinic Acid

Cardiomyocyte hypertrophy is a typical pathological feature in the late stage of heart failure. Rosmarinic acid (RA) has shown a certain effect on high glucose- (HG-) induced cardiomyocyte mitophagy and hypertrophy [154]. RA treatment increased PINK1 and Parkin pathway activation, promoted mitophagosome formation, inhibited ROS production, and restored mitochondrial activity. However, blocking Parkin activation led to inhibition of Parkin-mediated mitophagy and the protective effects of RA against HG-induced oxidative stress and cardiomyocyte hypertrophy was prevented. Therefore, we hypothesized that RA may protect rat cardiomyocytes by activating Parkin-mediated mitophagy and improving cardiomyocyte activity [155].

We previously discussed that ferulic acid has protected cardiomyocytes by regulating mitophagy; however, the specific regulatory mechanism has not been elucidated. According to in vitro experiments [156], mitophagy is a selective form of autophagy that can become hyperactivated in hypoxic/reoxygenated H9C2 cells. Ferulic acid can prevent heart damage by scavenging free radicals and affecting oxidative injury. Ferulic acid attenuated apoptosis, reduced mitochondrial dysfunction induced by hypoxia/reoxygenation injury, inhibited ROS overproduction and ATP depletion, restored mitochondrial membrane potentials, and reduced the binding of mitochondria to lysosomes. Ferulic acid downregulated the PINK1/Parkin pathway. It can further antagonize rapamycin-induced mitophagy activation. These experimental results suggest that ferulic acid can protect cardiomyocytes from ischemia-induced hypoxia by inhibiting PINK1/Parkin-dependent mitophagy. This is consistent with the earlier results that PINK1/Parkin-dependent mitophagy has selective bidirectional regulation.

#### 5.2.5. Shenmai Injection

In addition to the extracts and active ingredients of natural medicinal plants, Chinese herbal compounds and injections can also protect myocardial tissues and improve the activity of myocardial cells [157]. These drugs also play important regulatory roles in the protection and treatment of heart failure. Shenmai Injection (SMI) is a CFDA-approved herbal injection [157]. In an ethnopharmacology study, Yan et al. [158] found that SMI could regulate cardiomyocyte mitophagy and mitochondrial dynamics. Pretreatment with SMI significantly increased PINK/Parkin activity and mitophagy, regulated mitochondrial dynamics, inhibited excessive mitochondrial fission, increased the level of mitochondrial fusion, inhibited H/R-induced dysregulation of mitochondrial calcium homeostasis, and maintained mitochondrial structure and energy metabolism. Simultaneously, it inhibited cytoplasmic Ca^+^ overload, restored the increased MMP (*ΔΨ*m), regulated the abnormal opening of mPTP in mitochondria, relieved severely damaged mitochondrial respiratory function, and improved the survival rate of cardiomyocytes.

### 5.3. Natural Medicinal Plants Protect Cardiomyocytes through FUNDC1-Mediated Receptor-Dependent Mitophagy

Along with research on the regulation of PINK1/Parkin-related mitophagy, investigating the effects of natural medicinal plants on the FUNDC1 pathway has become popular in recent years.

#### 5.3.1. Baicalein

Baicalein is a natural flavonoid extracted from the medicinal plant Scutellaria baicalensis and has various pharmacological activities, including antiviral and antioxidative effects [159]. Baicalein has been found to protect cardiac hypertrophy in vivo and in vitro [160]. Baicalein administration can attenuate ISO-induced myocardial hypertrophy and restore cardioprotection [161]. In cardiomyocytes, ISO treatment increased excess accumulation of ROS and inhibited FUNDC1-mediated mitophagy. Baicalein (30 *μ*M) pretreatment directly bound to the transcription factor FOXO3a, promoting its transcriptional activity and transactivation of catalase and FUNDC1 while increasing mitophagy expression, inhibiting the overproduction and accumulation of ROS, and attenuating ISO-induced cardiac hypertrophy.

#### 5.3.2. Irisin

In the progression of heart failure pathology, the inflammatory response and the influence of the inflammatory environment are also important factors in developing myocardial injury, hypertrophy, and fibrosis [162]. Mitophagy can effectively regulate mitochondrial and intracellular homeostasis and maintain mitochondrial energy metabolism and cardiomyocyte activity. It also modulates mitochondria-mediated activation of the NLRP3 inflammasome and the inflammatory response of cardiomyocytes, thereby inhibiting the progression of heart failure [163].

FUNDC1 and mitophagy levels were significantly decreased in lipopolysaccharide- (LPS-) stimulated cardiomyocytes, but irisin treatment significantly increased FUNDC1-mediated mitophagy. It also improved glucose metabolism and significantly reduced LPS by increasing the activities of GPX/SOD, inhibiting the excessive generation of ROS, activation of caspase-3/-9, and LPS-stimulated cardiomyocyte apoptosis. However, after FUNDC1 knockdown in cardiomyocytes, the regulatory mechanisms of irisin on oxidative stress, mitochondrial energy metabolism, and mitophagy in cardiomyocytes were eliminated, and the caspase-3/caspase-9-mediated apoptosis was suppressed. These results suggest that the regulatory effects of irisin on cardiomyocyte activity and mitochondrial energy metabolism are mediated by FUNDC1 [164].

#### 5.3.3. Berberine

In addition to FUNDC1, BNIP3 belongs to the receptor-dependent mitophagy type. As mentioned previously, BBR may regulate mitophagy through the PINK/Parkin pathway and protect cardiomyocytes. Another study on berberine [165] found that BBR pretreatment can regulate the expression of autophagy-related proteins and induce cell proliferation and autophagosome formation. Luciferase reporter gene and chromatin immunoprecipitation (ChIP) assays indicated that BBR could mediate BNIP3 expression by enhancing the binding of HIF-1*α* to the BNIP3 promoter, increasing the level of mitophagy, and restoring the MMP (*ΔΨ*m) level in cardiomyocytes after hypoxia. In vivo experiments also showed that BBR intervention can reduce the area of myocardial injury, inhibit myocardial cell apoptosis, and significantly reduce the activities of myocardial enzymes (CK-MB, LDH, and aspartate transaminase). In addition, after BNIP3 knockdown, the regulatory effects of BBR on mitophagy and mitochondrial homeostasis were eliminated, and its protective effect on cardiomyocyte injury was further blocked. These experimental results suggest that the protective effect of BBR on cardiomyocytes may occur through receptor-dependent mitophagy mediated by BNIP3.

#### 5.3.4. Danqi Pills

The Danqi Pill (DQP) is composed of the dried roots of Salvia miltiorrhiza and Panax notoginseng [166] and has good therapeutic effects in the clinical treatment of heart failure. The related research reveals that this Chinese herbal compound regulates FUNDC1-mediated mitophagy to protect cardiomyocytes [167]. In animal experiments, a rat model of heart failure was established to evaluate the efficacy of DQP through ligation of the left anterior descending coronary artery. Interventions were performed using different doses of DQP. A moderate dose of DQP significantly improved cardiac function and inhibited cardiomyocyte apoptosis in rats with heart failure. Immunofluorescence staining showed that DQP increased the fluorescent colocalization of LC3B and the mitochondrial membrane TOM complex (TOM20). Coimmunoprecipitation experiments showed that DQP increased the colocalization of FUNDC1 with ULK1 and PGAM5, further restoring mitophagy. Wang et al. further modeled cardiomyocytes induced by glucose deprivation and reperfusion to elucidate the underlying mechanism of DQP. DQP protected against potential mitochondrial membrane damage, reduced cardiomyocyte apoptosis, reduced mitochondrial ROS levels, and increased ATP levels.

Other molecular biology experiments revealed that DQP increased the interaction between FUNDC1 and LC3B, and knockdown of FUNDC1 inhibited this interaction and mitophagy. The experimental results suggest that DQP may improve mitochondrial energy metabolism by improving FUNDC1-mediated mitophagy through the interaction mechanism of ULK1 and PGAM5, thereby treating heart failure.

## 6. Conclusion

The pathology of heart failure is complex. Mechanisms may involve injuries from myocardial ischemia, myocardial hypertrophy, and myocardial fibrosis, including but not limited to factors like hyperglycemia, hyperlipidemia, obesity, aging, genetics, and sex as well as living environment. Abnormal intracellular signal transduction, abnormal vascular endothelial metabolism, abnormal cell growth cycles, and necroptosis are related mechanisms that can be regulated by mitophagy and mitochondrial oxidative stress. Mitochondrial dysfunction has been described as an impaired ability of the mitochondria to transform nutrient substrates in response to changes in cellular energy demand. This, consequently, causes dysregulated homeostasis in cardiomyocytes, dysfunctional energy metabolism, and severe oxidative stress damage. Dysfunctional mitochondrial fractions are separated from the healthy mitochondrial network and degraded by mitophagy. However, mitophagy is insufficient for eliminating fragmented mitochondria with reduced membrane potentials, and mitochondria initiate caspase-3/caspase-9 or caspase-12 to promote cardiomyocyte apoptosis.

All relevant studies evaluating the active ingredients of natural medicinal plants have emphasized the functional importance of mitochondria in maintaining the function and homeostasis of cardiomyocytes. However, it is difficult to clarify the specific pharmacodynamic material basis and action mechanisms of traditional Chinese medicine compounds, compound preparations, and single active ingredients of traditional Chinese medicines. In this review, we clarified that mitophagy is a “double-edged sword,” and moderately regulated mitophagy can benefit the mitochondria and mitochondrial energy metabolism of cardiomyocytes. At the same time, excessive mitophagy can negatively impact the mitochondrial energy metabolism of cardiomyocytes. Cardiomyocyte mitochondria can have deleterious effects. Therefore, further discussion should be carried out regarding the administration dosage, timing, and staging, to exploit the favorable side of mitophagy. This involves maximizing the therapeutic advantages and the potential of natural medicinal plant active ingredients while reducing toxicity and synergy. Starting with various aspects, such as optimizing the detection of mitophagy and combining this with disease animal models, we can provide more useful evidence for treating heart failure with traditional Chinese medicine. Chinese medicine can regulate mitophagy and provide new ideas and methods for the clinical treatment of heart failure. This review explains the mechanisms of natural medicinal plants with different targets for protecting the myocardium and cardiomyocytes from different aspects of mitophagy (including receptor-dependent and receptor-independent processes), which can be used for future basic experimental research on heart failure.

## Data Availability

The data used to support the findings of this study are available from the corresponding author upon request.

## Abbreviations

HF: Heart failure
CHM: Natural medicinal plants (Chinese herbal medicine)
I/R: Ischemia/reperfusion
H/R: Hypoxia/reoxygenation
mtDNA: Mitochondrial DNA
ROS: Reactive oxygen species
MDA: Malondialdehyde
SOD: Superoxide dismutase
GSH: Glutathione
CAT: Catalase
GPX: Glutathione peroxidases
BAX: BCL2-associated X
NF-*κ*B: Nuclear factor *κ*-light-chain-enhancer of activated B cells
Drp1: Dynamin-related protein 1
Fis1: Mitochondrial fission 1 protein
TOM20: Mitochondrial membrane (TOM complex)
Mfn1: Mitofusin-1
MMP: Mitochondrial membrane potential
LPS: Lipopolysaccharides.
